# Associations of Cognitive Impairment with Putative Glymphatic-Related Imaging Indices and Cortical Atrophy in Cerebral Amyloid Angiopathy

**DOI:** 10.3390/biomedicines14061217

**Published:** 2026-05-28

**Authors:** Fumine Tanaka, Toshiaki Taoka, Maki Umino, Ryota Kogue, Hidehiro Ishikawa, Yuichiro Ii, Akihiro Shindo, Hajime Sakuma, Masayuki Maeda

**Affiliations:** 1 Department of Radiology, Mie University School of Medicine, Tsu 514-8507, Mie, Japanr-kogue@med.mie-u.ac.jp (R.K.);; 2 Department of Innovative Biomedical Visualization (iBMV), Nagoya University Graduate School of Medicine, Nagoya 464-8550, Aichi, Japan; 3 Department of Neurology, Mie University School of Medicine, Tsu 514-8507, Mie, Japan; hidehiro-i@med.mie-u.ac.jp (H.I.); a-shindo@med.mie-u.ac.jp (A.S.); 4 Department of Neuroimaging and Pathophysiology, Mie University School of Medicine, Tsu 514-8507, Mie, Japan; 5 Department of Neuroradiology, Mie University School of Medicine, Tsu 514-8507, Mie, Japan

**Keywords:** cerebral amyloid angiopathy, glymphatic system, cortical atrophy, DWI-ALPS index, cognitive impairment, MRI

## Abstract

**Purpose**: The aim of this study was to compare the contributions of putative glymphatic-related imaging indices—diffusion-weighted image analysis along the perivascular space (DWI-ALPS) index and choroid plexus volume (CPV)—and total cortical gray matter volume (TCGMV) to cognitive function in cerebral amyloid angiopathy (CAA). **Methods**: Forty-four CAA patients and 22 controls underwent 3.0T MRI. Cognitive function was assessed by the Mini-Mental State Examination (MMSE). The mean DWI-ALPS index, CPV/intracranial volume (ICV), and TCGMV/ICV were compared between groups; hierarchical multivariable regression and mediation analyses evaluated MMSE correlates. **Results**: Compared with controls, CAA showed a lower mean DWI-ALPS index and TCGMV/ICV (both adjusted *p* < 0.05), whereas CPV/ICV did not differ significantly after adjustment. In hierarchical multivariable regression analysis, mean DWI-ALPS index was associated with MMSE before adjustment for TCGMV/ICV (*p* = 0.022), but this association was attenuated after TCGMV/ICV was added to the model (*p* = 0.665). CPV/ICV was not associated with MMSE in either model, whereas TCGMV/ICV was independently associated with MMSE (*p* = 0.013). Exploratory mediation analysis suggested an indirect association between mean DWI-ALPS and MMSE via TCGMV/ICV (indirect: *p* = 0.023; direct: *p* = 0.720). **Conclusions**: Cortical atrophy appeared to be the strongest imaging correlate of cognitive impairment in CAA, while the association between DWI-ALPS and MMSE in multivariable models was attenuated after accounting for cortical gray matter volume. The ALPS index may provide indirect information on glymphatic-related pathways, but its biological specificity in CAA requires cautious interpretation because ALPS measurements may be influenced by underlying microstructural alterations in white matter.

## 1. Introduction

Cerebral amyloid angiopathy (CAA) is a typically age-related disease that causes cognitive impairment due to amyloid beta (Aβ) accumulation in leptomeningeal and cortical arteries [1,2]. In addition, CAA is frequently observed in patients with Alzheimer’s disease (AD) [1,2]. CAA is a subtype of small vessel disease (SVD) that causes various vascular lesions, such as cerebral microbleeds (CMBs), cerebral superficial siderosis (cSS), white matter hyperintensity (WMH), and enlarged perivascular space in the centrum semiovale (CSO-PVS) on MRI [2]. In contrast, PVSs in the basal ganglia (BG) are more likely associated with hypertension (HT)-related small vessel disease [3,4]. Recent guidelines emphasize that careful evaluation of CAA- and SVD-related hemorrhagic markers on MRI is particularly important when assessing amyloid-related abnormalities in patients receiving anti-amyloid-β antibody treatment [5]. Furthermore, a previous study demonstrated that the SVD score derived from these MRI scans is associated with cognitive function [6].

Another study of hereditary CAA with minimal parenchymal Aβ showed that global cortical atrophy contributes to memory function [7]. In contrast, sporadic CAA involves both vascular and neurodegenerative processes. Although hippocampal atrophy is not specific to AD, most typical AD cases show prominent hippocampal involvement [8,9]. AD-signature regions are affected in both early AD and CAA, representing a shared substrate for neurodegeneration [10,11]. Thus, AD-signature cortical thickness may serve as a surrogate marker reflecting convergent vascular and neurodegenerative pathologies.

Recently, the brain clearance systems, especially the glymphatic system, have garnered attention. Since 2017, multiple studies have suggested that the diffusion tensor image analysis along the perivascular space (DTI-ALPS) index may partly reflect glymphatic-related fluid transport [12,13,14,15,16,17,18]. However, DTI is not routinely included in standard clinical MRI protocols because of its relatively long acquisition time. In contrast, diffusion-weighted image (DWI) analysis is widely available. To enhance clinical applicability, diffusion-weighted image analysis along the perivascular space (DWI-ALPS) was proposed, retaining fundamental diffusion principles of DTI-ALPS [19]. A more recent report showed that the ALPS index can be affected not only by diffusivity along perivascular spaces but also by the surrounding white matter architecture [20]. Thus, fractional anisotropy (FA) of the corpus callosum (CC), which has a relatively uniform fiber architecture and fewer crossing fibers, should be assessed alongside the ALPS index [20] using DTI with sufficient diffusion-encoding directions [21,22,23]. Choroid plexus volume (CPV) has also been proposed as a complementary neurofluid-related marker [24,25]. Recent CAA studies have also examined CPV together with ALPS and other glymphatic-related markers, although its pathological significance in CAA remains unclear [26]. Accordingly, in this study, we use the term “putative glymphatic-related imaging indices” to refer to MRI-derived measures, including the DWI-ALPS index and CPV, that have been proposed to be associated with glymphatic-related pathways but do not directly measure glymphatic flow. Although the glymphatic system is reportedly involved in cognitive impairment [27], the extent to which glymphatic-related alterations contribute to cognitive function in CAA remains largely unknown. In CAA, Aβ accumulates along the vascular basement membranes, reflecting impairment of intramural periarterial drainage (IPAD), a key pathway for perivascular clearance that is functionally linked to the glymphatic system [28,29,30]. Although there is no established radiological method for evaluating the activity of IPAD, glymphatic-related imaging indices may indirectly reflect dysfunction.

Therefore, we hypothesized that both putative glymphatic system alterations and global cortical atrophy contribute to cognitive impairment in patients with CAA. The aim of this study was to compare the relative contributions of putative glymphatic-related imaging indices, including the DWI–ALPS index and CPV, and TCGMV to cognitive function in patients with CAA. As a secondary aim, we examined whether microstructural white matter damage, assessed by DTI-derived FA of the CC, influences the interpretation of the ALPS index in CAA.

## 2. Materials and Methods

### 2.1. Subjects

**Figure 1:**
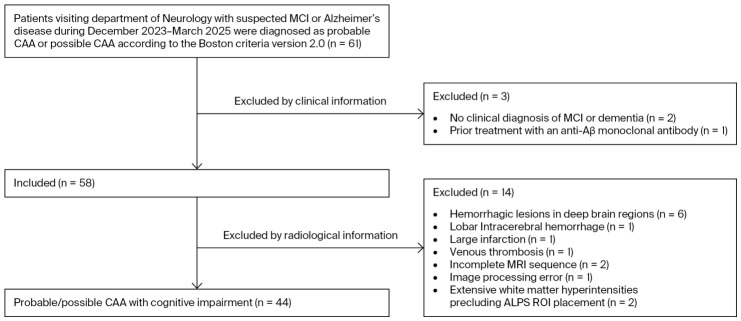
Flowchart of patient selection for the CAA group.

This study was approved by the ethics committee of Mie University, and the requirement for written informed consent was waived because of the retrospective study design. All procedures were conducted according to the principles of the World Medical Association’s Declaration of Helsinki. From December 2023 to March 2025, patients who visited the Department of Neurology with suspected mild cognitive impairment or AD and were diagnosed with probable or possible CAA based on the Boston criteria version 2.0 were included [31] (Figure 1). The exclusion criteria for the CAA group were as follows: (a) absence of a clinical diagnosis of either mild cognitive impairment (MCI) or dementia by neurologists (*n* = 2); (b) prior treatment with an anti-Aβ monoclonal antibody (*n* = 1); (c) hemorrhagic lesions in deep brain regions (basal ganglia, thalamus, or brainstem) (*n* = 6); (d) lobar intracerebral hemorrhage larger than 10 mm (*n* = 1); (e) large infarction (*n* = 1); (f) venous sinus thrombosis (*n* = 1); (g) incomplete MRI sequences (*n* = 2); (h) image processing error (*n* = 1); and (i) extensive white matter hyperintensities precluding ALPS region-of-interest (ROI) placement (*n* = 2). Consequently, 44 patients with sporadic CAA were included in the CAA group (16 males and 28 females; age range, 62–87 years; mean age 74.91 ± 6.56 years). Twenty-nine patients with probable CAA and 15 patients with possible CAA were included in the CAA group. Age- and sex-matched control patients (7 males and 15 females; age range 60–88 years; mean age 71.73 ± 7.05 years) were included if they underwent brain MRI for evaluation of cerebral aneurysms measuring 1–15 mm in diameter (*n* = 15), screening for metastases (*n* = 3), screening for middle cerebral artery (MCA) stenosis (*n* = 2), screening for olfactory dysfunction (*n* = 1), or screening for tinnitus (*n* = 1). This control cohort represents a clinically heterogeneous group, rather than cognitively normal community-based controls, although similar pragmatic control selection has been adopted in previous DWI-ALPS studies [32]. The exclusion criteria for the control group included hemorrhagic lesions larger than 10 mm, large territorial or cortico-subcortical infarctions, tumors, severe cerebral arterial stenosis, and more than three cerebral microbleeds (CMBs), based on a previous 1.5T susceptibility-weighted imaging study indicating that healthy controls do not exhibit more than three CMBs [33]. Cases with a history of clinically diagnosed cognitive impairment, neurological disease, or brain surgery were excluded. None of the control patients met the Boston criteria version 2.0 for probable or possible CAA.

### 2.2. Cognitive Function Assessments

The MMSE (range, 0–30; lower scores indicate worse global cognition) of patients with CAA was assessed by a neurologist or a speech therapist to quantify cognitive function, and was performed within a mean of 7.18 ± 15.95 days of MRI acquisition (range, 19 days before to 58 days after the imaging). Cognitive assessments were not systematically available for the control group.

### 2.3. MRI Protocol

All images were acquired using a 3.0T clinical MRI scanner, Ingenia (Philips Healthcare, Best, The Netherlands), with a 32-channel coil. The following DWI sequence parameters were used: repetition time (TR) = 6159 ms; echo time (TE) = 93 ms; b values = 0, 1000 s/mm^2^; phase-encoding direction = anterior-to-posterior (AP); FOV = 220 × 220 mm; matrix = 112 × 112; slice thickness = 3 mm; slice number = 48; scan time = 1 min 14 s; and axial imaging plane on the anterior commissure–posterior commissure (AC-PC) line. Diffusion-encoding gradients were aligned with the imaging plane. Other imaging protocols, including DTI, 3D T1-weighted imaging, susceptibility-weighted image-phase (SWI-P), and 3D fluid-attenuated inversion recovery (FLAIR), are documented in Supplementary Materials S1 (Table S1). DTI was additionally performed in a subset of participants: 4 controls and 17 patients with CAA.

### 2.4. Image Processing

#### 2.4.1. Mean DWI-ALPS Index

The mean DWI-ALPS index was obtained for all participants using a method described elsewhere [19]. Diffusivity maps of each subject were obtained along the x-axis (right-left; ADCx), y-axis (anterior–posterior; ADCy), and z-axis (inferior–superior; ADCz). No correction for susceptibility distortion or eddy currents and motion was applied to the DWI data. We calculated the DWI–ALPS index as the ratio of the mean of the x-axis ADC in the projection fiber area (ADCxproj) and the x-axis ADC in the association fiber area (ADCxassoc) to the mean of the y-axis ADC in the projection fiber area (ADCyproj) and the z-axis ADC in the association fiber area (ADCzassoc) using Equation (1):

(1) $$DWI-ALPS\;index=\frac{mean\;(ADCxproj,\;ADCxassoc)}{mean\;(ADCyproj,\;ADCzassoc)}$$

**Figure 2:**
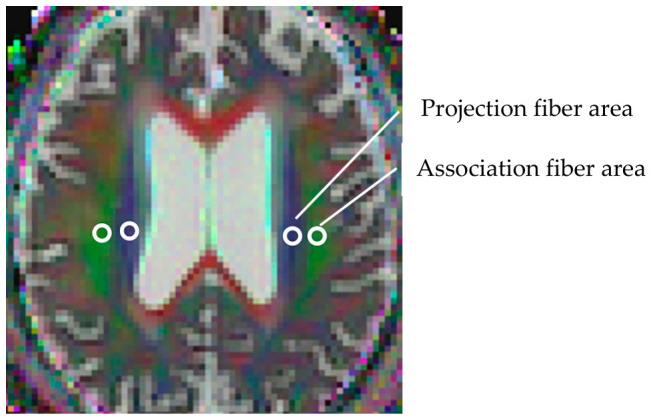
Method for ALPS index measurement.

Generation of ADC images and composite images and calculation of the DWI–ALPS index were performed using ImageJ version 1.54d software (National Institutes of Health, Bethesda, MD, USA) (Figure 2). ROIs were manually placed by two experienced neuroradiologists (F.T. and R.K.) and then checked against 3D FLAIR and SWI-P to confirm no overlap with visible WMH or CMB. Representative examples of ROI placement across different CAA lesion burdens are shown in Figure S1. The ALPS index derived from DWI was calculated for the projection and association areas in the right hemisphere (R-ALPS index) and left hemisphere (L-ALPS index), and the average of the right and left values was used for statistical analysis. This approach follows previous DTI-ALPS studies in CAA, in which hemispheric values were combined to derive a single ALPS measure [27]. In addition, in a large autopsy study, CAA pathology was assessed by combining pathological findings across cortical regions to yield an overall burden measure, without explicit hemispheric stratification, and greater overall CAA burden was associated with cognitive impairment [34].

On the slice including the body of the lateral ventricle of the color-coded ADC/FA map, in which the x-, y-, and z-axes were displayed in red, green, and blue, respectively, we identified the projection fiber and association fiber areas. Circular regions of interest (ROIs) were manually placed bilaterally in the projection fiber areas and bilaterally in the association fiber areas (four ROIs in total: right and left projection fibers, and right and left association fibers). The DWI-ALPS index and DTI-ALPS index were measured using Equations (1) and (2):

(2) $$\mathrm{DTI}\text{-}\mathrm{ALPS}\;\mathrm{index}\;=\;\frac{mean\;(Dxxproj,Dxxassoc)}{mean\;(Dyyproj,Dzzassoc)}$$

The mean DTI- and DWI-ALPS indices were calculated as the averages of the right and left hemispheric values.

#### 2.4.2. Mean DTI-ALPS Index

The mean DTI-ALPS index was calculated in a subset of the overall study cohort consisting of participants who underwent DTI (4 controls and 17 patients with CAA). The detailed process is described in Supplementary Materials S2 (Method S1) and a previous study [12].

#### 2.4.3. Corpus Callosum DTI FA Measurement

FA values of the CC genu, body, and splenium were extracted from DTI data obtained from participants who underwent DTI (4 controls and 17 patients with CAA). Tract-based spatial statistic (TBSS) processing and region-of-interest extraction were performed using the corresponding regions from the Johns Hopkins University ICBM white-matter labels atlas [35], as detailed in Supplementary Materials S2 (Method S2).

#### 2.4.4. Structural Volume and Cortical Thickness Measurements

All structural volume measurements were derived from 3D T1-weighted images using FreeSurfer and the computational anatomy (CAT)12 toolbox of Statistical Parametric Mapping (SPM) 12 as follows. Bilateral CPV, hippocampal volume (HV) [25,36], WMH volume (WMHV) [37,38], and total cortical gray matter volume (TCGMV) [39,40] were calculated. All volumetric measures were normalized by intracranial volume (ICV) to obtain CPV/ICV, HV/ICV, WMHV/ICV, and TCGMV/ICV. An AD-signature cortical thickness measure was calculated. The process is described in detail in Supplementary Materials S2 (Method S3).

### 2.5. Radiological Visual Assessments

**Figure 3:**
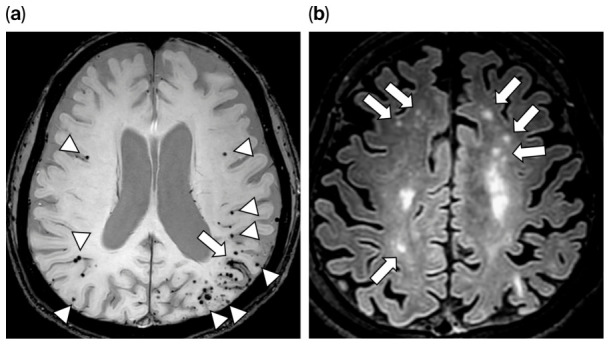
A representative CAA case in a 74-year-old man (MMSE score, 27). (**a**) SWI-P shows multiple CMBs (arrowheads) in both hemispheres, and cSS (arrow) in the sulcus of the left parietal lobe. (**b**) 3D-FLAIR shows WMH multi-spot patterns (arrows) in both hemispheres.

**Figure 4:**
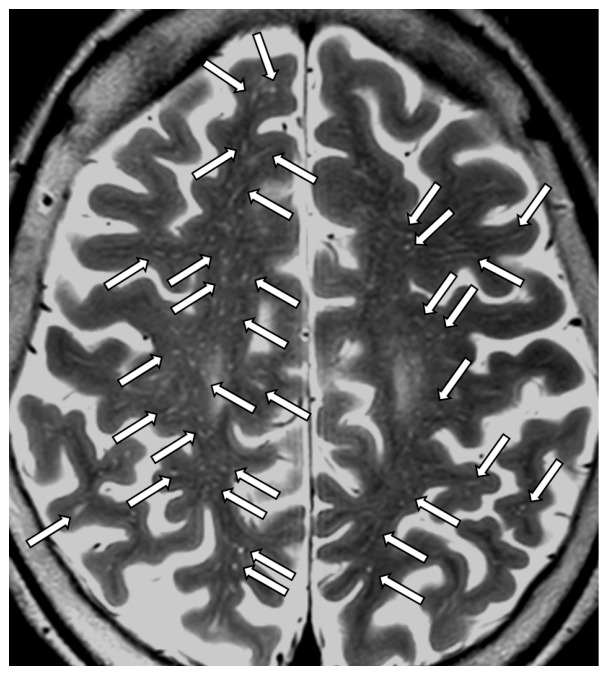
A representative CAA case in an 87-year-old woman (MMSE score, 28). The T2-weighted image shows enlarged CSO-PVS (arrows) in both hemispheres (grade 4).

Visual imaging findings were evaluated according to the Standards for Reporting Vascular Changes on Neuroimaging (STRIVE)-2 and the Boston criteria version 2.0, with reference to previously published studies; detailed definitions and grading procedures are provided in Supplementary Materials S2 (Method S4) [31,41,42,43,44,45,46,47]. A representative case is shown in Figure 3 and Figure 4.

### 2.6. Statistical Analysis

All statistical analyses were performed using R version 4.2.1 (R Foundation for Statistical Computing, Vienna, Austria). Inter-rater reliability for visually assessed MRI markers was evaluated by two neuroradiologists (R.K. and F.T.). Quadratic weighted Cohen’s κ was used for ordinal variables (lobar CMB, deep CMB, cSS, CSO-PVS, BG-PVS). For binary WMH patterns (multi-spots and posterior-dominant), agreement was assessed using observed agreement, both positive and negative. Inter-rater reliability for lobar lacune counts and mean DTI-ALPS and DWI-ALPS indices was assessed using the intraclass correlation coefficient (ICC), applying an absolute-agreement, two-way random-effects model; single-measure ICC was used for the ALPS indices. Inter-rater agreement was evaluated using quadratic weighted Cohen’s κ, interpreted as follows: <0.20 slight, 0.21–0.40 fair, 0.41–0.60 moderate, 0.61–0.80 substantial, and >0.80 almost perfect agreement [48]. For ICC, values <0.50 were considered poor, 0.50–0.75 moderate, 0.75–0.90 good, and >0.90 excellent [49].

After assessing normality with the Shapiro–Wilk test, group comparisons regarding demographic data, MRI visual findings, and MRI quantitative measurements were performed as appropriate. Group differences in the mean DTI-ALPS index and DTI-derived FA of the CC subregions (genu, body, and splenium) were explored descriptively between patients with CAA (*n* = 17) and controls (*n* = 4), given the limited sample size of the control group. Age- and sex-adjusted analyses were applied to non-DTI imaging markers using multivariable regression models. DTI metrics were compared between groups using unadjusted analyses because of the limited sample size; these analyses were considered exploratory. Because five controls were included for clinical screening purposes, including metastasis screening (*n* = 3) and mild MCA stenosis (*n* = 2), sensitivity analyses with additional adjustment for complication status were performed. Correlation analyses between the mean DTI-ALPS index and DTI-derived FA of the CC were conducted separately within each group, as appropriate. To account for multiple comparisons across CC subregions, *p* values were adjusted using the Benjamini–Hochberg false discovery rate (FDR) correction. Given the particularly small number of controls, correlation analyses were primarily interpreted within the CAA group.

In the CAA group (*n* = 44), hierarchical multivariable linear regression analyses were performed with MMSE as the dependent variable. Age and sex were included first, followed by mean DWI-ALPS index and CPV/ICV, with TCGMV/ICV added subsequently. Separate models replaced TCGMV/ICV with HV/ICV or AD-signature cortical thickness. Multicollinearity was assessed using variance inflation factors (VIFs). Robust regression analyses were performed using M-estimation (rlm, MASS). As an additional sensitivity analysis, each imaging marker was entered individually into the base model to examine its incremental contribution to MMSE.

Exploratory mediation analysis was performed using the “lavaan” package in R to examine whether TCGMV/ICV mediated the relationship between the Mean DWI-ALPS index and MMSE scores. This analysis was exploratory and based on cross-sectional data; therefore, it was not intended to establish causal or temporal relationships. Consistent with the regression analysis, this model was conducted in the full CAA cohort (*n* = 44) and adjusted for age, sex, and CPV/ICV. All continuous variables were standardized to Z-scores. The significance of the indirect effect was determined using bootstrapping with 5000 resamples. *p* < 0.05 was considered statistically significant.

## 3. Results

### 3.1. MMSE Assessments

The MMSE scores were obtained for all 44 patients with CAA, and the average MMSE score was 24.39 ± 2.97, ranging from 15 to 29 (Table 1).

### 3.2. Inter-Rater Reliability of Mean DTI/DWI-ALPS Index and Visual Assessments

Inter-rater reliability was good for both the DTI-ALPS index (0.841, 95% CI 0.292–0.967, *p* = 0.009) and the DWI-ALPS index (0.795, 95% CI 0.516–0.914, *p* < 0.001) (Table 2). Furthermore, the inter-rater reliability for visual MRI markers was good to almost perfect (Supplementary Materials S3 Table S2).

### 3.3. Correlation Analysis Between Mean DWI-ALPS Index and Mean DTI-ALPS Index

Pearson’s correlation analysis (*n* = 21) demonstrated a strong positive association between mean DWI-ALPS and mean DTI-ALPS indices (r = 0.888, 95% CI 0.739–0.954, *p* < 0.001) (Supplementary Materials S4, Figure S2).

### 3.4. Between-Group Differences in Demographic Data

The demographic data of the CAA and control groups are presented in Table 1. There were no significant differences in age, sex, and the prevalence of HT, dyslipidemia (DL), or diabetes mellitus (DM) between the groups (*p* > 0.05).

### 3.5. Between-Group Differences in Radiological Visual Assessments and MRI Quantitative Measurements

Among visual MRI markers, cSS grade was higher and the WMH multi-spot pattern was more frequent in the CAA group (adjusted *p* < 0.05), whereas no other markers showed significant between-group differences (adjusted *p* > 0.05) (Table 3). Among quantitative measurements, the mean DWI-ALPS index was significantly lower in patients with CAA than in controls (1.30 ± 0.12 vs. 1.41 ± 0.17; adjusted *p* = 0.011). TCGMV/ICV was also significantly lower in the CAA group (0.25 ± 0.02 vs. 0.27 ± 0.02; adjusted *p* < 0.001). In addition, HV/ICV was significantly lower in the CAA group than in the controls (4.27 ± 0.52 vs. 5.06 ± 0.47 × 10^−3^; adjusted *p* < 0.001), and AD-signature area cortical thickness was also lower (2.48 ± 0.21 vs. 2.65 ± 0.12 mm; adjusted *p* = 0.002). CPV/ICV and WMHV/ICV did not show significant between-group differences after adjustment (adjusted *p* ≥ 0.05) (Table 4 and Supplementary Materials S4, Figure S3). In the sensitivity analyses additionally adjusted for complication status (age, sex, and complication), the overall pattern of the results was similar; however, the association for the WMH multi-spot pattern was attenuated and no longer statistically significant (adjusted *p* = 0.075) (Supplementary Materials S3, Table S3). In a small DTI subset (*n* = 21), exploratory analyses suggested that the mean DTI-ALPS index was lower in the CAA group than in the controls (1.30 ± 0.15 vs. 1.64 ± 0.10, *p* = 0.0015). FA of the CC body was also lower in the CAA group (0.60 ± 0.04 vs. 0.65 ± 0.03, *p* = 0.020), whereas no significant differences were observed in the genu or splenium (both *p* > 0.05) (Supplementary Materials S3, Table S4).

### 3.6. Exploratory Correlation Analysis Between Mean DTI-ALPS Index and DTI-FA in the CC

In the controls, no significant correlations were observed between the mean DTI-ALPS index and FA in any corpus callosum subregion after FDR correction. In patients with CAA, the mean DTI-ALPS index showed positive correlations with FA in the CC body (r = 0.621, FDR-adjusted *p* = 0.023) and splenium (r = 0.567, FDR-adjusted *p* = 0.026), whereas the correlation with FA in the CC genu was not significant after FDR adjustment (Supplementary Materials S4, Figure S4).

### 3.7. Hierarchical Multivariable Regression and Sensitivity Analysis

In the CAA group (*n* = 44), hierarchical multivariable regression analyses showed that the addition of mean DWI-ALPS index and CPV/ICV to the demographic model was associated with a trend toward improved model fit (ΔR^2^ = 0.117, *p* = 0.068) (Table 5), with mean DWI-ALPS index showing a significant association with MMSE (B = 8.498, *p* = 0.022), whereas CPV/ICV was not associated with MMSE (B = −0.001, *p* = 0.997). Further inclusion of TCGMV/ICV significantly improved the model (ΔR^2^ = 0.120, *p* = 0.013), and TCGMV/ICV was independently associated with MMSE (B = 68.178, *p* = 0.013), whereas the association of the mean DWI-ALPS index was attenuated and no longer significant (B = 1.830, *p* = 0.665). CPV/ICV remained unassociated with MMSE after TCGMV/ICV was added (B = 0.068, *p* = 0.862). In models accounting for potential AD co-pathology, HV/ICV did not significantly improve model fit (ΔR^2^ = 0.018, *p* = 0.361) and was not associated with MMSE. In contrast, AD-signature cortical thickness significantly improved the model (ΔR^2^ = 0.163, *p* = 0.003) and was independently associated with MMSE (B = 6.05, *p* = 0.003). Multicollinearity was not observed in any model (all VIFs < 2.0) (Supplementary Materials S3 Table S5). Robust regression analyses yielded consistent results (Supplementary Materials S3 Table S6), with both TCGMV/ICV (B = 73.075, 95% CI: 6.902 to 125.353) and AD-signature cortical thickness (B = 5.903, 95% CI: 2.675 to 13.001) remaining significantly associated with MMSE. In contrast, HV/ICV was not associated with MMSE (B = 912.778, 95% CI: −1693.5 to 3211.9). In the sensitivity analyses, the addition of individual imaging markers resulted in only minimal increases in explained variance (ΔR^2^: 0.000–0.053), and none were significantly associated with MMSE. In contrast, TCGMV/ICV remained consistently associated with MMSE across all models (and multicollinearity was not observed in any model (all VIFs < 3.5)) (Supplementary Materials S3 Table S7).

### 3.8. Exploratory Statistical Mediation Analysis

**Figure 5:**
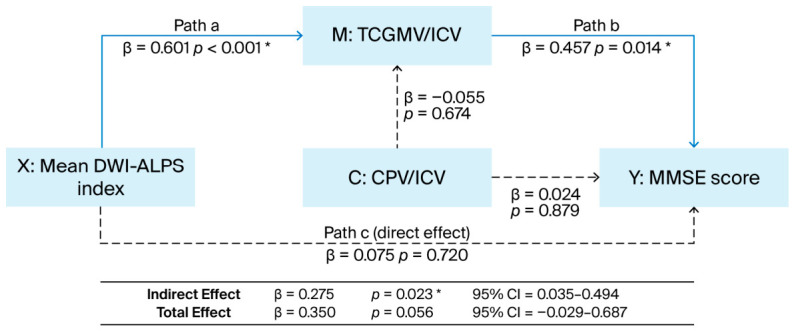
Exploratory statistical mediation analysis of the association between the mean DWI-ALPS index and MMSE score via TCGMV/ICV. The exploratory mediation model was adjusted for age, sex, and CPV/ICV (*n* = 44). Standardized coefficients (β) and *p*-values are provided for each path. Solid blue lines indicate statistically significant paths (*p* < 0.05), while dashed lines indicate non-significant paths. * *p* < 0.05, significant difference.

An exploratory statistical mediation analysis (*n* = 44) showed that the mean DWI-ALPS index was significantly associated with TCGMV/ICV (path a: β = 0.601, *p* < 0.001) (Figure 5), and TCGMV/ICV was significantly associated with MMSE (path b: β = 0.457, *p* = 0.014). The indirect association of the mean DWI-ALPS index on MMSE via TCGMV/ICV was significant (β = 0.275, 95% CI: 0.035–0.494, *p* = 0.023), whereas the direct association was not (path c: β = 0.075, *p* = 0.720). CPV/ICV was not significantly associated with either TCGMV/ICV (β = −0.055, *p* = 0.674) or MMSE (β = 0.024, *p* = 0.879).

## 4. Discussion

In this study, patients with CAA showed lower mean DWI-ALPS index and lower TCGMV/ICV than the controls. In the multivariable regression analyses with MMSE as the dependent variable, TCGMV/ICV showed the strongest association with MMSE across models, whereas putative glymphatic-related imaging indices were not significantly associated in this dataset. To our knowledge, few studies have directly compared putative glymphatic-related imaging indices and global cortical gray matter volume within the same cohort of patients with CAA. The present study provides a comparative evaluation of these imaging correlates in relation to cognitive function. However, interpretation of the group differences requires caution, as the control group represents a clinically heterogeneous cohort rather than cognitively normal individuals, and disease specificity cannot be established.

In our study, a lower ALPS index was observed in patients with CAA, which is consistent with a previous study [27]. In contrast to our findings, the same previous study has reported a direct association between the ALPS index and cognition [27]. This discrepancy may reflect methodological differences between DWI-ALPS and DTI-ALPS, and limited statistical power due to the small cohort. The relatively weak association of DWI-ALPS with MMSE in CAA may partly reflect the predominant role of IPAD, rather than a primary contribution of glymphatic pathways. Pathological studies have shown that Aβ deposition preferentially involves superficial vessels and extends toward deeper segments, supporting IPAD dysfunction as a central mechanism in CAA [50]. In multivariable regression analysis, the association between the DWI-ALPS index and MMSE was attenuated after adding TCGMV/ICV to the model, suggesting that cortical gray matter volume may partly account for the DWI-ALPS–MMSE relationship. The exploratory statistical mediation analysis suggested a potential indirect association between the DWI-ALPS index and MMSE via cortical atrophy, with no significant direct association. These findings are broadly consistent with previous reports suggesting associations among glymphatic-related imaging indices, structural brain changes, and cognitive impairment [51]. From a practical perspective, these findings suggest that reduced ALPS values in patients with CAA should be interpreted together with structural markers, particularly cortical gray matter volume, rather than being considered in isolation as specific evidence of glymphatic dysfunction. However, because the mediation analysis was based on cross-sectional data, the interpretation remains tentative, and causal or temporal relationships cannot be established. In addition, the ALPS index may be influenced by white matter microstructure and imaging-related factors, including susceptibility-induced distortion related to nearby hemorrhagic lesions, even when regions with visible lesions are avoided. Therefore, the mediation result should be interpreted as exploratory and should not be regarded as evidence of a biological glymphatic pathway or causal sequence.

CPV/ICV tended to be higher in CAA than controls, but the difference was not statistically significant. CPV has been proposed as a complementary marker of neurofluid homeostasis, and previous studies have reported an association between CPV enlargement and cognitive impairment. However, recent CAA studies suggest that CPV findings are less consistent than ALPS-related findings; therefore, its pathological significance in CAA remains uncertain and warrants further investigation [26]. Similarly, CSO-PVS grade did not differ between groups, which was inconsistent with a previous report [3]. This visual method may be more susceptible to subjectivity than quantitative measurements [52].

In this study, TCGMV/ICV was significantly lower in the CAA group than controls and was associated with MMSE, indicating that global cortical atrophy is an important imaging correlate of cognitive impairment in CAA. While HV/ICV was not associated with MMSE, AD-signature cortical thickness was. Given that CAA has been shown to cause cortical thinning in regions overlapping with AD-signature areas, these findings suggest that cognitive impairment in CAA may be driven by both CAA-related cortical neurodegeneration and co-existing AD pathology [9,11]. This is consistent with recent evidence linking CAA severity to cortical atrophy and AD pathology, rather than hippocampal atrophy [53].

From a pathophysiological perspective, several CAA-related mechanisms may underlie this cortical gray matter loss. Cortical microinfarcts (CMIs), a characteristic feature of CAA, are important contributor to cortical thinning [54]. CMIs are associated with CAA severity and preferentially occur in cortical regions vulnerable to vascular dysfunction [55,56,57]. Aβ deposition in cortical and leptomeningeal vessels impairs vascular smooth muscle cell function and disrupts cerebral autoregulation, leading to chronic cortical and subcortical hypoperfusion [56,58,59]. This hemodynamic vulnerability, together with blood–brain barrier breakdown and associated inflammatory responses, exacerbates microvascular injury and promotes the development of CMIs [57,59]. The cumulative burden of CMIs may contribute to cortical thinning through neuronal loss and network disruption [7,11,60].

In our exploratory analysis, DTI-ALPS index showed positive correlations with FA in the CC body and splenium in patients with CAA, suggesting that the ALPS index may reflect not only perivascular fluid dynamics but also underlying white matter microstructure. This interpretation is consistent with studies showing that the ALPS index can be affected by local white matter microstructure, especially in the CC [20,61]. This finding highlights an important interpretive constraint. Previous DTI studies have also reported white matter alterations involving the CC in CAA [62], which may reflect “distant effects” of cortical CAA pathology [62]. However, given the small cohort, particularly in the control group, these findings should be interpreted with caution and considered preliminary, requiring validation in larger cohorts. Notably, DTI-derived FA was used solely as a complementary measure to characterize corpus callosum microstructural integrity and does not imply that DTI is required for clinical use of the ALPS index.

There are several limitations to this study. First, pathological confirmation of CAA was not available. In addition, amyloid positron emission tomography (PET), tau PET, cerebrospinal fluid (CSF) biomarkers, and plasma biomarkers were not obtained; therefore, the influence of concomitant AD pathology could not be fully excluded [63]. Although HV/ICV and AD-signature cortical thickness were examined as imaging markers potentially related to AD pathology, these surrogate measures cannot definitively distinguish CAA-related cortical atrophy from concomitant AD pathology. Moreover, because the CAA cohort was recruited from patients referred for suspected MCI or AD, it may have been enriched for patients presenting with cognitive complaints or concomitant AD-related pathology. Therefore, the generalizability of our findings to broader CAA populations, including asymptomatic or hemorrhage-predominant CAA, may be limited. Future studies including more diverse CAA populations are needed to validate and extend our findings. Second, in the DTI analyses, the control group was particularly small, limiting statistical power; therefore, these findings require validation in larger cohorts. Third, cognitive assessment was limited to the MMSE in the CAA group, and detailed neuropsychological tests, including assessments of executive function, processing speed, and attention, were not available. Therefore, the present findings may primarily reflect global cognitive impairment rather than CAA-specific cognitive dysfunction. In addition, objective cognitive assessments were not available for the control group. Because of the retrospective design, MMSE assessments and MRI examinations were not performed on the same day, and interval effects cannot be excluded; prospective studies with same-day and detailed cognitive assessments are warranted. Fourth, the control group was clinically heterogeneous, although none of the control patients met the Boston criteria version 2.0 for probable or possible CAA. Moreover, the control group included individuals with fewer than four CMBs, which may nevertheless better represent a real-world population of community-dwelling older adults, among whom CMBs are commonly observed [33,64,65,66]. Therefore, the specificity of between-group comparisons should be interpreted with caution. Fifth, distortion and eddy-current corrections were not applied to the DWI data. Because the primary ALPS analysis was based on limited-direction clinical DWI, the impact of conventional correction procedures may be modest in this context. In contrast, DTI data included multi-direction diffusion encoding, and correction procedures were applied in the DTI subset, which may have improved measurement accuracy. Although ROIs were placed according to the standard ALPS method in central white matter regions, which are not typical predilection sites for CAA-related lobar CMBs and where geometric distortion is generally minimal, residual distortion or susceptibility-related effects cannot be completely excluded. Sixth, manual ALPS ROI placement may introduce operator dependence, anatomical variability, and lesion-avoidance bias. Future studies should adopt more standardized approaches, such as standard-space registration, atlas-based ROI definition, or automated ROI placement, to improve reproducibility. Finally, this study had a cross-sectional design, and no longitudinal data were available. Therefore, the prognostic value of the ALPS index, its temporal progression, and its sensitivity to disease evolution or therapeutic intervention remain unknown. Future longitudinal studies are needed to determine whether combined assessment of ALPS-related measures and cortical atrophy can allow for predicting cognitive decline or monitoring disease progression in CAA.

## 5. Conclusions

Cortical atrophy appeared to be the strongest imaging correlate of cognitive impairment in CAA, while the association between DWI-ALPS and MMSE in multivariable models was attenuated after accounting for cortical gray matter volume. The ALPS index may provide indirect information on glymphatic-related pathways, but its biological specificity in CAA requires cautious interpretation because ALPS measurements may be influenced by underlying microstructural alterations in white matter.

## Figures and Tables

**Table 1:** Patients’ demographic data.

	CAA (*n* = 44)	Control (*n* = 22)	*p*	Statistical Analysis
Age, mean, years (range)	74.91 ± 6.56 (62–87)	71.73 ± 7.05 (60–88)	0.075	*t*-test
	Probable CAA (*n* = 29)			
	76.17 ± 6.51 (62–87)			
	Possible CAA (*n* = 15)			
	72.47 ± 6.14 (62–80)			
Sex, male (%)	16 (36.36)	7 (31.82)	0.715	Pearson χ^2^
HT (%)	17 (38.64)	13 (59.09)	0.116	Pearson χ^2^
DL (%)	18 (40.91)	4 (18.18)	0.065	Pearson χ^2^
DM (%)	6 (13.64)	1 (4.55)	0.258	Pearson χ^2^
Years of education, mean years (range)	12.39 ± 1.93 (9–16), Missing data *n* = 13	—	—	—
MMSE, mean score (range)	24.39 ± 2.97 (15–30)	—	—	—

Abbreviations: CAA, cerebral amyloid angiopathy; HT, hypertension; DL, dyslipidemia; DM, diabetes mellitus; MMSE, Mini-Mental State Examination. *p* < 0.05, significant difference. Years of education and MMSE data were not available in the control group.

**Table 2:** Inter-rater reliability of mean DTI/DWI-ALPS index.

Measure	Statistic	Value	95% CI	*p*	Interpretation
Mean DTI-ALPS index	ICC (2, 2)	0.841	0.292–0.967	0.009	Good
Mean DWI-ALPS index	ICC (2, 2)	0.795	0.516–0.914	<0.001 *	Good

Abbreviations: DTI, diffusion tensor image; DWI, diffusion-weighted image; ALPS, along the perivascular space; CI, confidence interval; ICC, intraclass correlation coefficient. * *p* < 0.05, significant difference.

**Table 3:** Between-Group Differences in Radiological visual assessments.

	CAA (*n* = 44)	Control (*n* = 22)	*p*	Adjusted *p*	Statistical Analysis
Lobar CMB grade (grade 0:1:2:3:4:5)	16:17:6:1:2:2	11:11:0:0:0:0	0.064	0.150	Mann–Whitney U/Ordinal logistic
Deep CMB grade (grade 0:1)	44:0	21:1	0.167	0.999	Mann–Whitney U/Binary logistic
cSS grade (none: focal: disseminated)	37:3:4	22:0:0	0.051	<0.001	Mann–Whitney U/Ordinal logistic
Lacunar, count (mean ± SD)	0.27 ± 1.34	0.00 ± 0.00	0.325	0.622	Mann–Whitney U/Linear model
CSO-PVS grade (grade 1:2:3:4)	9:22:12:1	10:5:7:0	0.250	0.206	Mann–Whitney U/Ordinal logistic
BG-PVS grade (grade 1:2:3:4)	33:10:1:0	19:3:0:0	0.283	0.331	Mann–Whitney U/Ordinal logistic
WMH multi-spot pattern (%)	41 (93.18)	15 (68.18)	0.012 *	0.048 *	Pearson χ^2^/Logistic
WMH posterior-dominant pattern (%)	17 (38.64)	3 (13.64)	0.037 *	0.104	Pearson χ^2^/Logistic

Abbreviations: CAA, cerebral amyloid angiopathy; CMB, cerebral microbleed; cSS, cortical superficial siderosis; WMH, white matter hyperintensity; PVS, perivascular space; CSO, centrum semiovale; BG, basal ganglia. * *p* < 0.05 was considered statistically significant. Adjusted *p* values were obtained from linear regression models adjusted for age and sex.

**Table 4:** MRI quantitative measurements.

Variable	CAA (*n* = 44)	Control (*n* = 22)	*p*	Adjusted *p*	Statistical Analysis
Mean DWI-ALPS index	1.30 ± 0.12	1.41 ± 0.17	0.003 *	0.011 *	Student t/Linear regression
CPV/ICV (×10^−3^)	2.58 ± 1.05	2.06 ± 0.67	0.008 *	0.078	Welch t/Linear regression
WMHV/ICV (×10^−6^)	6.45 ± 5.48	3.75 ± 2.62	0.054	0.144	Mann–Whitney U/Linear regression
TCGMV/ICV	0.25 ± 0.02	0.27 ± 0.02	<0.001 *	<0.001 *	Student t/Linear regression
HV/ICV (×10^−3^)	4.27 ± 0.52	5.06 ± 0.47	<0.001 *	<0.001 *	Student t/Linear regression
AD-signature area cortical thickness (mm)	2.48 ± 0.21	2.65 ± 0.12	<0.001 *	0.002 *	Mann–Whitney U/Linear regression

Abbreviations: CAA, cerebral amyloid angiopathy; DWI, diffusion-weighted image; ALPS, analysis along the perivascular space; CPV, choroid plexus volume; ICV, intracranial volume; WMHV, white matter hyperintensity volume; TCGMV, total cortical gray matter volume; HV, hippocampal volume; AD, Alzheimer disease. * *p* < 0.05 was considered statistically significant. Adjusted *p* values were obtained from linear regression models adjusted for age and sex.

**Table 5:** Hierarchical multivariable linear regression analysis for MMSE in CAA (*n* = 44).

Variable	Model 1: Demographic	Model 2: +Mean DWI-ALPS Index + CPV/ICV	Model 3A: +TCGMV/ICV	Model 3B-1: +HV/ICV	Model 3B-2: +AD-Signature Area Cortical Thickness
	B (95% CI), *p*	B (95% CI), *p*	B (95% CI), *p*	B (95% CI), *p*	B (95% CI), *p*
Mean DWI-ALPS index	—	8.498 (1.297 to 15.699), 0.022 *	1.830 (−6.662 to 10.323), 0.665	7.439 (−0.146 to 15.024), 0.054	5.462 (−1.334 to 12.258), 0.112
CPV/ICV	—	−0.001 (−0.839 to 0.836), 0.997	0.068 (−0.716 to 0.851), 0.862	0.034 (−0.809 to 0.877), 0.936	−0.001 (−0.758 to 0.756), 0.998
TCGMV/ICV	—	—	68.178 (15.168 to 121.188), 0.013 *	—	—
HV/ICV	—	—	—	804.713 (−958.486 to 2567.913), 0.361	—
AD-signature thickness	—	—	—	—	6.053 (2.147 to 9.959), 0.003 *
Age	0.128 (−0.009 to 0.264), 0.065	0.146 (0.011 to 0.280), 0.034 *	0.151 (0.026 to 0.277), 0.020 *	0.152 (0.017 to 0.288), 0.029 *	0.154 (0.033 to 0.276), 0.014 *
Sex (female)	−0.576 (−2.412 to 1.259), 0.530	−0.949 (−2.759 to 0.860), 0.295	−1.294 (−3.005 to 0.417), 0.134	−0.960 (−2.774 to 0.855), 0.291	−0.672 (−2.317 to 0.973), 0.413
Model Summary					
	Model 1	Model 2	Model 3A	Model 3B-1	Model 3B-2
R^2^	0.091	0.208	0.328	0.226	0.371
Adjusted R^2^	0.047	0.127	0.240	0.124	0.289
ΔR^2^	—	+0.117	+0.120	+0.018	+0.163
*p* (ΔR^2^)	—	0.068	0.013 *	0.361	0.003 *

Abbreviations: MMSE, Mini-Mental State Examination; CAA, cerebral amyloid angiopathy; B, unstandardized regression coefficient; CI, confidence interval; DWI-ALPS, diffusion-weighted imaging–analysis along the perivascular space; CPV, choroid plexus volume; ICV, intracranial volume; TCGMV, total cortical gray matter volume; HV, hippocampal volume; AD, Alzheimer disease. Sex was included as a binary variable (male as reference). * *p* < 0.05, significant difference.

## Data Availability

The data presented in this study are available on request from the corresponding author. The data are not publicly available due to privacy restrictions.

## Supplementary Materials

The following supporting information can be downloaded at: https://www.mdpi.com/article/10.3390/biomedicines14061217/s1, Table S1: Imaging protocol; Table S2: Inter-rater reliability of the visual assessments; Table S3: Sensitivity analyses with additional adjustment for clinical screening status (metastasis and middle cerebral artery stenosis) for visual and quantitative imaging findings; Table S4: Mean DTI-ALPS index and DTI-FA in the CC; Table S5: Variance inflation factors for hierarchical multivariable linear regression models; Table S6: Robust regression analysis for MMSE in CAA (*n* = 44); Table S7: Sensitivity analysis of imaging markers added to the base model; Method S1: Calculation of the DTI-ALPS index; Method S2: Corpus callosum fractional anisotropy analysis; Methods S3: Volumetric and cortical thickness analyses; Methods S4: Visual assessment of CAA-related imaging findings; Figure S1: Representative ROI placement for DWI ALPS index in cases with different CAA lesion burdens; Figure S2: Correlation analysis between mean DWI ALPS index and mean DTI ALPS index (4 controls and 17 participants with CAA); Figure S3: Group comparisons of mean DWI ALPS index and MRI quantitative measurements; Figure S4: Correlation analysis between mean DTI ALPS index and FA in the CC (4 controls and 17 participants with CAA).

## Abbreviations

Aβ	Amyloid beta
AD	Alzheimer’s disease
ALPS	Along the perivascular space
CAA	Cerebral amyloid angiopathy
CC	Corpus callosum
CMB	Cerebral microbleed
CPV	Choroid plexus volume
CSO	Centrum semiovale
cSS	Cortical superficial siderosis
DTI	Diffusion-tensor image
DWI	Diffusion-weighted image
FA	Fractional anisotropy
ICV	Intracranial volume
IPAD	Intramural periarterial drainage
MCI	Mild cognitive impairment
MMSE	Mini-Mental State Examination
PVS	Perivascular space
SVD	Small vessel disease
TCGMV	Total cortical gray matter volume
WMHV	White matter hyperintensity volume
