# Supplementary material for: Associations of Cognitive Impairment with Putative Glymphatic-Related Imaging Indices and Cortical Atrophy in Cerebral Amyloid Angiopathy
Source: Biomedicines. 2026 May 28;14(6):1217. doi: 10.3390/biomedicines14061217 (PMC13296348; doi:10.3390/biomedicines14061217)
Supplement: Supplementary file 1 [file biomedicines-14-01217-s001.zip › Supplementary materials S1.260421.pdf]

Supplementary materials S1

Table S1. Imaging protocol

(a) Diffusion-tensor imaging parameters

TR	8530 ms
TE	79 ms
diffusion-encoding directions	15 axes
b value	0, 800s/mm^2
phase encode direction	posterior-to- anterior (PA)
FOV	220×220mm
matrix	112×112
slice thickness	2 mm
slice number	72
scan time	3 minutes 6 seconds

(b) Other imaging sequences parameters

	3D T1-weighted imaging	SWI-P	3D FLAIR (CAA group)	3D FLAIR (Control group)
FOV,mm	270	230	250	250
RFOV, %	90.62	82.3	100	100
Matrix	288	384	256	256
Recon matrix	384	768	480	480
Scan %	99.9	78.3	72.1	72.1
Slice	200	140	350	350
Slice thickness, mm	0.94	2→1	1.14→0.57	1.14→0.57
TR, ms	8.2	31	6000	6000
TE, ms	4.6	7.2<br>Delta TE 6.2	380	380
ACQ voxel	0.94×0.74×0.94	0.6×0.6×2.0	0.98×1.06×1.14	0.98×1.06×1.14
Recon voxel	0.70×0.70×0.94	0.3×0.3×1.0	0.52×0.52×0.57	0.52×0.52×0.57
NSA	1	1	2	2

	Compressed SENSE 3<br>TFE factor 260<br>TI 1100ms<br>FA 10	Compressed SENSE 4<br>Flow compensation yes	Compressed SENSE 8<br>TI 2000ms<br>Refocusing FA 40<br>Fat suppression SPIR<br>TSE factor 203	SENSE (P=2.6, S=2.6)<br>TI 2000ms<br>Refocusing FA 40<br>Fat suppression SPIR<br>TSE factor 203
Scan time	3 minutes 15 seconds	3 minutes 16 seconds	4 minutes 6 seconds	4 minutes 42 seconds
