# Supplementary material for: Associations of Cognitive Impairment with Putative Glymphatic-Related Imaging Indices and Cortical Atrophy in Cerebral Amyloid Angiopathy
Source: Biomedicines. 2026 May 28;14(6):1217. doi: 10.3390/biomedicines14061217 (PMC13296348; doi:10.3390/biomedicines14061217)
Supplement: Supplementary file 1 [file biomedicines-14-01217-s001.zip › Supplementary materials S2.260522.pdf]

Supplementary materials S2

Method S1: Calculation of the DTI-ALPS index

Mean DTI-ALPS index was obtained using FMRIB Software Library version 6.0.5.2 (FSL; Oxford Centre for Functional MRI of the Brain, Oxford, UK; www. fmrib.ox.ac.uk/fsl). The processing was described elsewhere [12]. Diffusion tensor fitting was performed using FSL without susceptibility distortion correction or eddy-current and motion correction (no–TopUp, no–Eddy) to obtain direction-specific tensor-derived diffusivities along the x-, y-, and z-axes (Dx, Dy, and Dz). ROI placement was performed using the same procedure as that used for the DWI-ALPS index.

Method S2: Corpus callosum fractional anisotropy analysis

FA values of the corpus callosum (CC) body derived from both DTI (available in 17 participants with CAA and 7 controls) were processed using TBSS implemented in the Oxford FMRIB Software Library (FSL; http://fsl.fmrib.ox.ac.uk/fsl). After eddy current– and motion-induced distortion correction of the diffusion-weighted data using FSL (eddy), brain extraction was performed on the b0 image to generate a brain mask. Diffusion tensors were fitted using dtifit to generate individual FA maps. TBSS was applied for voxelwise white matter analysis. Eddy-corrected FA maps were nonlinearly registered to

the FMRIB58 FA template in Montreal Neurological Institute (MNI) space, skeletonized, and projected onto a mean FA skeleton using a threshold of FA > 0.20.

For region-of-interest analyses, mean FA values of the CC genu, body, and splenium were extracted by intersecting the TBSS FA skeleton with the corresponding regions from the Johns Hopkins University ICBM white-matter labels atlas, and averaging skeletonized FA values within each region [35].

Methods S3: Volumetric and cortical thickness analyses

Bilateral CPV and HV were calculated using the Segmentation Based on Adaptive Multi-Scale and Expectation Maximization (SAMSEG) method [25, 36] , part of the FreeSurfer 7.3.2 (http:// surfer. nmr. mgh. harva rd. edu/). After the analysis, we confirmed misdetection or overdetection of choroid plexus. The CPV fraction (CPV/ICV) and HV fraction (HV/ICV) were defined as volumes divided by ICV that calculated by recon-all pipeline on Freesurfer 7.4.0.

WMH volume was calculated using the computational anatomy (CAT)12 toolbox (C. Gaser, Structural Brain Mapping Group, Jena University Hospital, Jena, Germany; http://dbm.neuro.uni-jena.de/cat/) Statistical Parametric Mapping 12 software (Wellcome Trust Center for Neuroimaging, London, UK; http://www.

fil.ion.ucl.ac.uk/spm/software/spm12/) [37, 38]. Specifically, WMH volume was derived through the default CAT12 automated tissue segmentation pipeline based on T1-weighted images. This process utilizes Adaptive Maximum A Posterior (AMAP) segmentation and a Partial Volume Estimation (PVE) model to identify local white matter hyperintensities—which appear as relative hypointensities on T1-weighted images—as a distinct tissue class without the need for additional FLAIR-based tools [38]. After the analysis, we confirmed misdetection or overdetection of WMH by referencing the FLAIR images. The WMH fraction was defined as WMH divided by ICV that calculated by recon-all pipeline on Freesurfer 7.4.0. Total cortical gray matter volume (TCGMV) was calculated with FreeSurfer 7.4.0 using the default recon-all pipeline [39, 40]. The TCGMV fraction was defined as TCGMV divided by ICV that calculated by recon-all pipeline on Freesurfer 7.4.0. The AD-signature cortical thickness was defined as the mean cortical thickness across bilateral entorhinal, fusiform, inferior temporal, middle temporal, inferior parietal, and precuneus regions derived from the FreeSurfer recon-all pipeline, yielding a single composite measure.

Methods S4: Visual assessment of CAA-related imaging findings

Visual imaging findings were evaluated according to the Standards for Reporting Vascular

Changes on Neuroimaging (STRIVE)-2 and the Boston criteria version 2.0 [31, 41]. The number of CMB, defined as hypointense foci measuring less than 10mm, was separately counted in the lobar and deep regions, which included the putamen, caudate nucleus, thalamus, brain stem, on SWI-P [41]. We created a grading system ranging from 0 to 5 as follows; grade 0, none; grade 1, 1-5 counts; grade 2, 6-10 counts; grade 3, 11-20 counts; grade 4, 21-50 counts; grade 5, more than 50 counts. Because SWI is more sensitive to CMBs than conventional T2*-weighted imaging, we anticipated higher lesion counts and therefore designed higher grades (4 and 5) to mitigate a ceiling effect [42, 43]. Cerebellar microbleed was not counted in this study. The number of cSS, defined as a linear hypointense signal in the sulci, occasionally extending into the gyri, was counted on SWI-P [41]. We categorized cSS according to previous studies as follows; none (no involvement of sulci), focal (involvement of 1-3 sulci), disseminated (involvement of 4 or more sulci) [44]. There was no case with cSS in the cerebellum. The number of lacune, defined as a round or ovoid infarction smaller than 15mm in diameter, and located in the lobar areas, was counted on 3D-FLAIR [41]. PVS were defined as fluid-filled spaces with signal intensity similar to cerebrospinal fluid on T2-weighted images, appearing round, ovoid, or linear along the course of penetrating vessels and measuring less than 3 mm in diameter [39]. PVS grade was evaluated at the slice of CSO, which was one centimeter

above the upper rim of the lateral ventricle body, and basal ganglia (BG), which included the maximum area of the BG as follows; grade 1, 1-10 counts; grade 2, 11-20 counts; grade 3, 21-40 counts; grade 4, more than 40 counts [45]. We assessed each PVS grade on both sides, and the higher grade between the two sides was used as the final PVS grade. The presence of WMH multi-spots pattern was evaluated, defined as 10 or more punctate WMH lesions located in the subcortical region of the cerebrum on 3D-FLAIR [46]. The presence of WMH posterior dominant pattern was also visually evaluated, defined as WMH located posterior to the lateral ventricles with a larger volume than those located anterior to the lateral ventricles [47].
