# Supplementary material for: Associations of Cognitive Impairment with Putative Glymphatic-Related Imaging Indices and Cortical Atrophy in Cerebral Amyloid Angiopathy
Source: Biomedicines. 2026 May 28;14(6):1217. doi: 10.3390/biomedicines14061217 (PMC13296348; doi:10.3390/biomedicines14061217)
Supplement: Supplementary file 1 [file biomedicines-14-01217-s001.zip › Supplementary materials S3.260522.pdf]

Supplementary materials S3

Table S2 Inter-rater reliability of the visual assessments

**Imaging marker**	**Scale / Type**	**Statistic**	**Value**	**Observed agreement (%)**	**Positive agreement (%)**	**Negative agreement (%)**	**PABAK**	**Interpretation**
Lobar CMB grade	Ordinal	Quadratic weighted Cohen's κ	0.980	NA	NA	NA	NA	Almost perfect
Deep CMB grade	Ordinal (0–1)	Quadratic weighted Cohen's κ	1.000	NA	NA	NA	NA	Almost perfect
cSS grade	Ordinal	Quadratic weighted Cohen's κ	1.000	NA	NA	NA	NA	Almost perfect
CSO-PVS	Ordinal	Quadratic weighted Cohen's κ	0.957	NA	NA	NA	NA	Almost perfect
BG-PVS	Ordinal	Quadratic weighted Cohen's κ	1.000	NA	NA	NA	NA	Almost perfect
Lobar lacune count	Count	ICC(2,2) (two-way random, absolute agreement, average measures)	0.889	NA	NA	NA	NA	Good
WMH pattern (multi-spots)	Binary	Observed agreement / positive & negative agreement /	0.826	94.74	96.77	85.71	0.895	Almost perfect

**Imaging marker**	**Scale / Type**	**Statistic**	**Value**	**Observed agreement (%)**	**Positive agreement (%)**	**Negative agreement (%)**	**PABAK**	**Interpretation**
		unweighted κ / PABAK						
WMH pattern (posterior-dominant)	Binary	Observed agreement / positive & negative agreement / unweighted κ / PABAK	1.000	100.0	100.0	100.0	1.000	Almost perfect

Abbreviations: PABAK, prevalence- and bias-adjusted kappa; CMB, cerebral microbleed; cSS, cortical superficial siderosis; CSO, centrum semiovale; PVS, perivascular space; BG, basal ganglia; WMH, white matter hyperintensity.

Table S3. Sensitivity analyses with additional adjustment for clinical screening status (metastasis and middle cerebral artery stenosis) for visual and quantitative imaging findings

	Adjusted p	Statistical analysis
Lobar CMB grade	0.234	Ordinal logistic (age, sex, clinical screening indication)
Deep CMB grade	0.999	Binary logistic (age, sex, clinical screening indication)
cSS grade	<0.001*	Ordinal logistic (age, sex, clinical screening indication)
CSO-PVS grade	0.433	Ordinal logistic (age, sex, clinical screening indication)
BG-PVS grade	0.562	Ordinal logistic (age, sex, clinical screening indication)
WMH multi-spots pattern	0.075	Binary logistic (age, sex, clinical screening indication)
WMH posterior-dominant pattern	0.225	Binary logistic (age, sex, clinical screening indication)
Lacunar, count	0.647	Linear model (age, sex, clinical screening indication)
Mean DWI-ALPS index	0.004*	Linear model (age, sex, clinical screening indication)
CPV/ICV	0.065	Linear model (age, sex, clinical screening indication)

	Adjusted p	Statistical analysis
WMHV/ICV	0.227	Linear model (age, sex, clinical screening indication)
TCGMV/ICV	<0.001*	Linear model (age, sex, clinical screening indication)
HV/ICV	<0.001*	Linear model (age, sex, clinical screening indication)
AD-signature area cortical thickness	0.004*	Linear model (age, sex, clinical screening indication)

Abbreviations: CMB, cerebral microbleed; cSS, cortical superficial siderosis; CSO-PVS, perivascular spaces in the centrum semiovale; BG-PVS, perivascular spaces in the basal ganglia; WMH, white matter hyperintensity; DWI-ALPS index, diffusion-weighted image analysis along the perivascular space index; CPV, choroid plexus volume; ICV, intracranial volume; WMHV, white matter hyperintensity volume; TCGMV, total cortical gray matter volume; HV, hippocampal volume; AD, Alzheimer disease.

*p < 0.05 was considered statistically significant.

Table S4. Mean DTI-ALPS index and DTI-FA in the CC

**Outcome**	**CAA (*n*=17)**	**Control (*n*=4)**	**p (unadjusted)**	**Statistical analysis**
Mean_DTI_ALPS_index	1.30 ± 0.15	1.64 ± 0.10	0.002*	t-test
DTI_CC_genu_FA	0.57 ± 0.05	0.62 ± 0.05	0.160	t-test
DTI_CC_body_FA	0.60 ± 0.04	0.65 ± 0.03	0.020*	t-test
DTI_CC_splenium_FA	0.68 ± 0.04	0.73 ± 0.04	0.068	t-test

Abbreviations: DTI, diffusion-tensor image; ALPS, along the perivascular space; FA, fractional anisotropy; CC, corpus callosum; CAA, cerebral amyloid angiopathy.

*p < 0.05 was considered statistically significant.

Table S5. Variance inflation factors for hierarchical multivariable linear regression models

Variable	Model 2: +Mean DWI -ALPS index +CPV/ICV	Model 3A: +TCGMV/ICV	Model 3B-1: +HV/ICV	Model 3B-2: +AD signature area cortical thickness
Mean DWI-ALPS index	1.060	1.690	1.169	1.156
CPV/ICV	1.101	1.106	1.110	1.101
TCGMV/ICV	—	1.741	—	—

Variable	Model 2: +Mean DWI -ALPS index +CPV/ICV	Model 3A: +TCGMV/ICV	Model 3B-1: +HV/ICV	Model 3B-2: +AD signature area cortical thickness
HV/ICV	—	—	1.156	—
AD-signature area cortical thickness	—	—	—	1.102
Age	1.064	1.065	1.075	1.066
Sex	1.061	1.088	1.061	1.073

VIF, variance inflation factor; DWI-ALPS, diffusion-weighted imaging–analysis along the perivascular space; CPV, choroid plexus volume; ICV, intracranial volume; TCGMV, total cortical gray matter volume; HV, hippocampal volume; AD, Alzheimer's disease.

Table S6 Robust regression analysis for MMSE in CAA (*n*=44)

Variable	Model 3A: +TCGMV/ICV	Model 3B-1: +HV/ICV	Model 3B-2: +AD signature area cortical thickness
	B (95% CI)	B (95% CI)	B (95% CI)
TCGMV/ICV	73.075 (6.902 to 125.353)	—	—
HV/ICV	—	912.778 (−1693.5 to 3211.9)	—
AD-signature area cortical thickness	—	—	5.903 (2.675 to 13.001)
Mean DWI-ALPS index	−0.726 (−9.374 to 13.008)	5.814 (−3.859 to 17.972)	3.096 (−3.971 to 13.115)
CPV/ICV	0.159 (−0.640 to 1.081)	0.088 (−0.921 to 1.126)	0.037 (−0.862 to 1.003)
Age	0.093 (−0.035 to 0.255)	0.098 (−0.048 to 0.288)	0.085 (−0.023 to 0.278)
Sex (female)	−1.658 (−3.166 to 0.386)	−1.275 (−3.160 to 1.013)	−1.028 (−2.951 to 1.244)

Abbreviations: MMSE, Mini-Mental State Examination; CAA, cerebral amyloid angiopathy; B, unstandardized regression coefficient; CI, confidence interval; DWI-ALPS, diffusion-weighted imaging–analysis along the perivascular space; CPV, choroid plexus volume; ICV, intracranial

volume; TCGMV, total cortical gray matter volume.

Sex was included as a binary variable (male as reference).

Table S7. Sensitivity analysis of imaging markers added to the base model

Variable	R^2	ΔR^2	TCGMV/ICV B	TCGMV/ICV p	Added variable B	Added variable p
Lobar CMB grade	0.360	0.031	68.125	0.012	0.465	0.186
BG PVS grade	0.382	0.053	57.549	0.034	1.489	0.082
Lacunar infarcts	0.336	0.008	66.848	0.016	0.220	0.506
WMH posterior dominant pattern	0.330	0.001	68.300	0.014	−0.246	0.780
cSS grade	0.334	0.005	70.743	0.012	0.379	0.586
WMHV/ICV	0.330	0.002	68.258	0.014	0.038	0.733
WMH multispots pattern	0.328	0.000	67.714	0.017	−0.137	0.938
CSO PVS grade	0.329	0.000	68.328	0.014	0.087	0.876

The base model was Model 3A, including age, sex, mean DWI-ALPS index, CPV/ICV, and TCGMV/ICV.

TCGMV/ICV B and p values represent the coefficient of TCGMV/ICV in each model.

Abbreviations: TCGMV, total cortical gray matter volume; ICV, intracranial volume; CMB, cerebral microbleed; cSS, cortical superficial siderosis; CSO, centrum semiovale; PVS, perivascular space; BG, basal ganglia; WMHV, white matter hyperintensity volume; WMH, white matter hyperintensity. $p < 0.05$, significant difference.
