# Supplementary material for: Associations of Cognitive Impairment with Putative Glymphatic-Related Imaging Indices and Cortical Atrophy in Cerebral Amyloid Angiopathy
Source: Biomedicines. 2026 May 28;14(6):1217. doi: 10.3390/biomedicines14061217 (PMC13296348; doi:10.3390/biomedicines14061217)

# Figure S1 **Representative ROI placement for DWI-ALPS index in cases with different CAA lesion burdens**

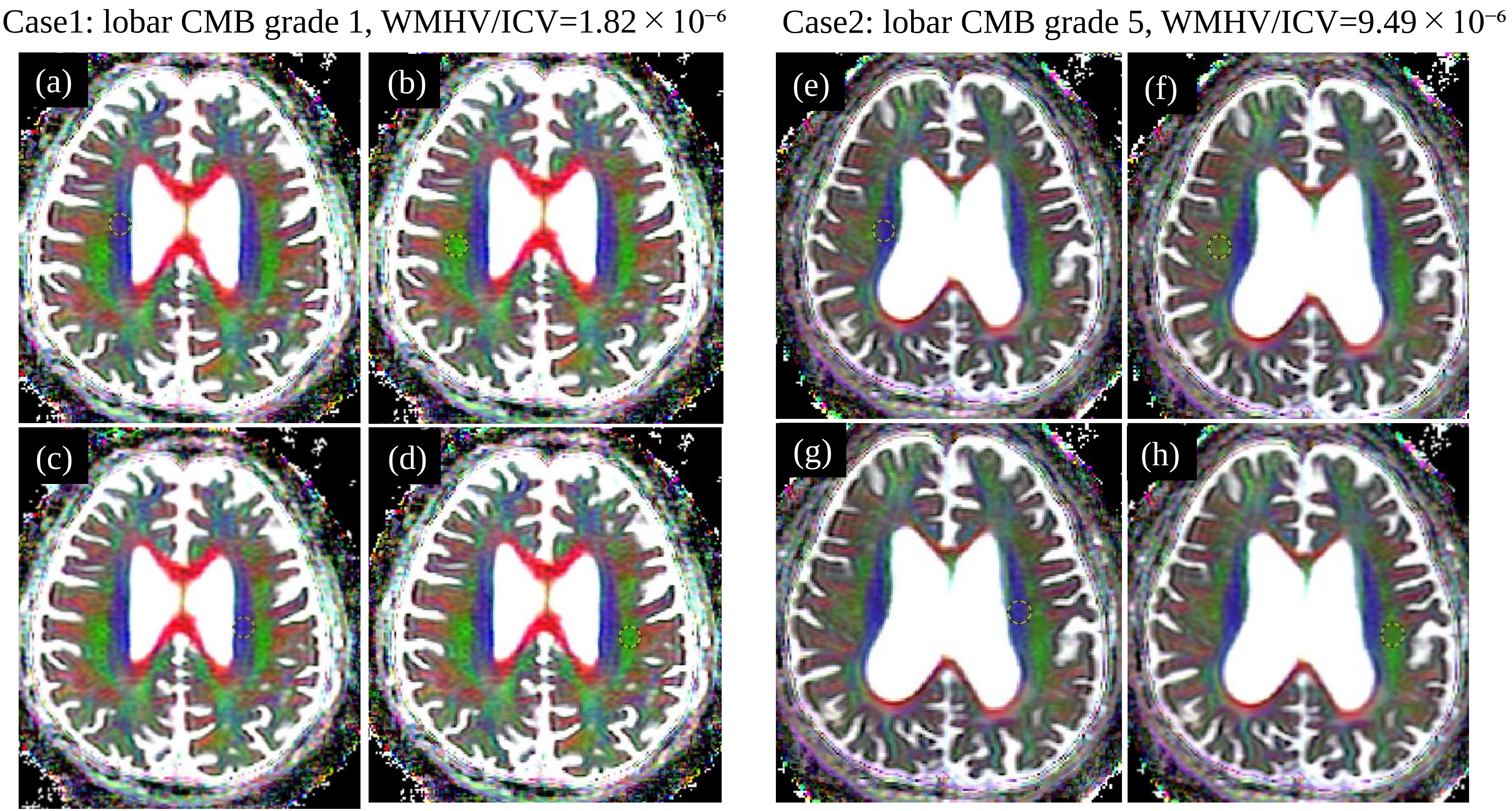

**Figure S2** **Correlation analysis between mean DWI-ALPS index and mean DTI-ALPS index (4 controls and 17 participants with CAA)**

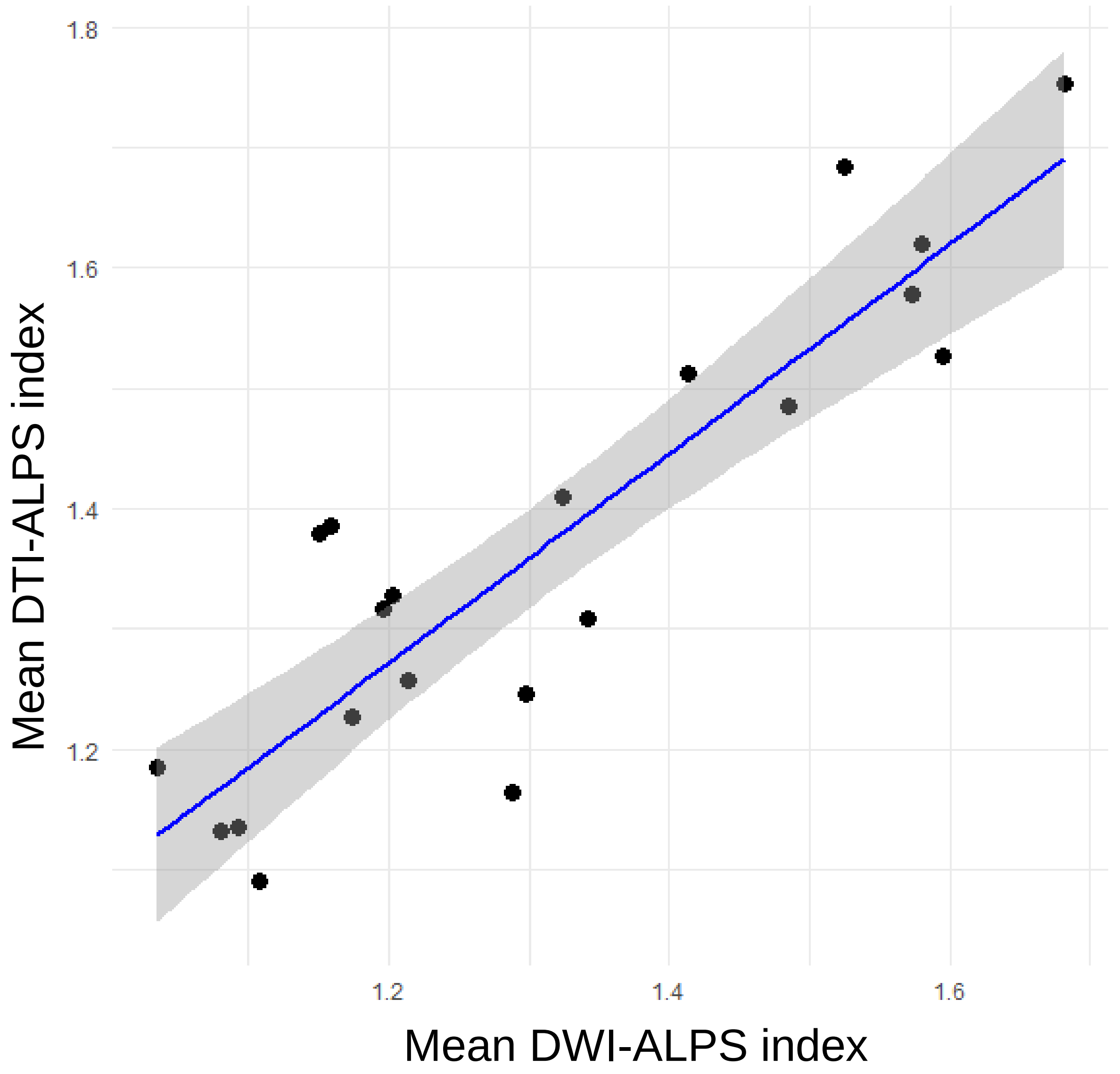

# Figure S3 Group comparisons of mean DWI-ALPS index and MRI quantitative measurements

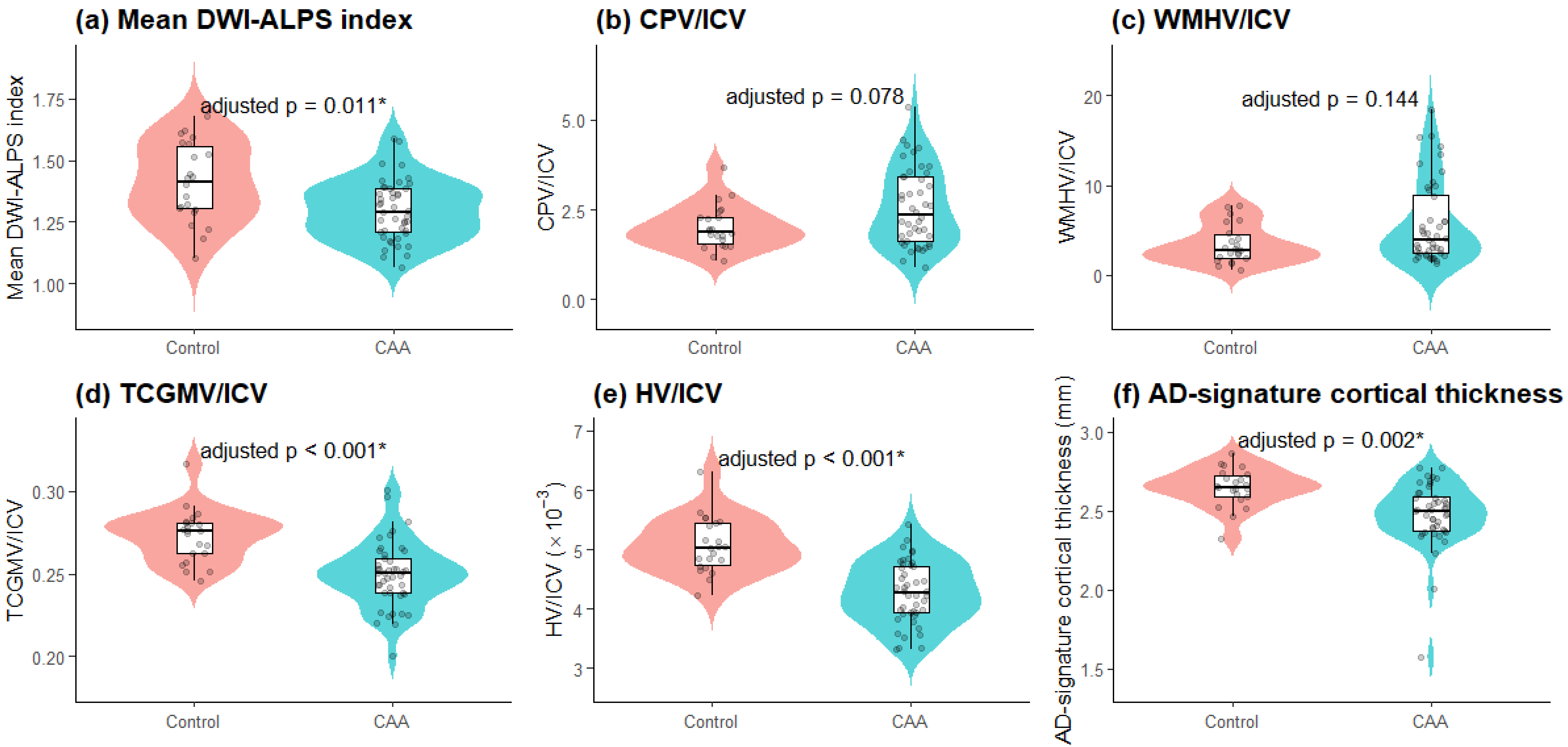

# Figure S4 Correlation analysis between mean DTI ALPS index and FA in the CC (4 controls and 17 participants with CAA)

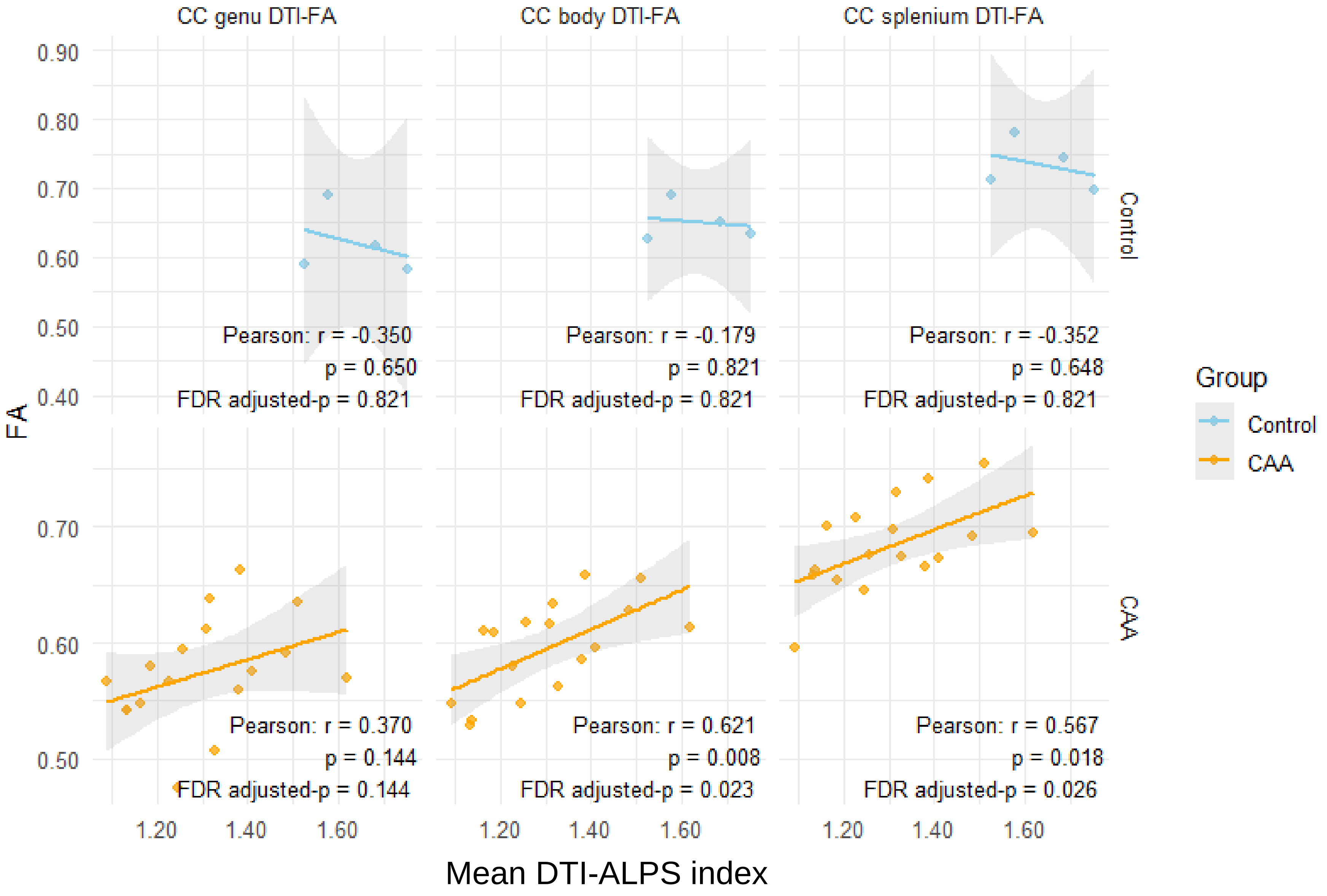

Supplement: Supplementary file 1 [file biomedicines-14-01217-s001.zip › Supplementary materials S4.260323.pdf]
